# 

**Published:** 1872-04

**Authors:** 


### INDEX OF SUBJECTS.

### ORIGINAL COMMUNICATIONS.

Effects of Railroad Travel upon the Health of Women........................ 193
Physical Signs of the Chest........................................ 206
Tape-Worm.................................................. 213
The Secretions as Guides to Treatment................................. 218
Treatment of Granular Ophthalmia, by the liberal use of Quinine............ 222
The treatment of Chronic Rheumatism.............................. 224
Vaccination papers; the Iodide of Ammonium and Sodium in Syphilis........ 227

### EXTRACTS AND GLEANINGS.

Cerebro-Spinal Meningitis, 228; Laxative Pill, 230; Bromide of Potassium in Spermatorrhœa, 231; Remarks on old Remedies, 232; Puerperal Convulsions, 233: Effects of Bromide Potassium, 234; A plan for facilitating reduction of Hernia—Flatulent Dyspepsia—Digitalis, 235; The use of Digitalis—Varicose Veins—Ulcerated Throat, etc., 236; Diabetes—Diet in Diabetes, 237; Suppurating Sores and Wounds incompatibles with perchloride Iron—An efficient Hæmatic, 238; Delirium Tremens—Antidote to liquor poisoning—Steam of Aqua Ammonia for Hooping-Cough, 239; New mode of administering Copabia—Stimulating Hypodermic Injections in Typhoid Fever, 240; Treatment of Headache, 253; Sulphocarbolate of Zinc in Otorrhœa, 254; On the utility of Calomel in Infantile Intestinal Affections, 255; To remove Tar, Pitch, Turpentine, etc., 256.

### EDITORIALS, REVIEWS, ETC.

To our Subscribers—Correction........................................ 241
Please Remember—Apologetic—Literary Periodicals,......................... 242
Peace in Georgia, at last........................................... 243
A Word with our Readers—The American Medical Association................ 244
The American Medical Association..................................... 245
Richmond and Louisville Medical Journal............................... 248

